# Incorporating *Helicobacter pylori* Infection: A Novel Model for Predicting Advanced Colorectal Neoplasia

**DOI:** 10.1111/hel.70130

**Published:** 2026-05-14

**Authors:** Xujin Chen, Dehua Tang, Ningjing Gao, Muhan Ni, Peng Yan, Zhanwen Ji, Xiang Xin, Cheng Yang, Lei Wang, Qiang Zhan

**Affiliations:** ^1^ Department of Gastroenterology The Affiliated Wuxi People's Hospital of Nanjing Medical University, Wuxi People's Hospital, Wuxi Medical Center, Nanjing Medical University Wuxi Jiangsu China; ^2^ Department of Gastroenterology Jiangsu Provincial Gastrointestinal Medical Innovation Center, Nanjing Drum Tower Hospital, Affiliated Drum Tower Hospital, Medical School of Nanjing University Nanjing Jiangsu China; ^3^ Department of Digestive Endoscopy Center The Affiliated Wuxi People's Hospital of Nanjing Medical University, Wuxi People's Hospital, Wuxi Medical Center, Nanjing Medical University Wuxi Jiangsu China

**Keywords:** advanced colorectal neoplasia, combined gastrointestinal tumor screening, gastric‐related indicators, *Helicobacter pylori* infection, risk prediction model

## Abstract

**Background and Aims:**

Early detection of advanced colorectal neoplasia (ACN) is critical for reducing colorectal cancer (CRC) incidence and mortality. Existing prediction models exhibit limited discriminative power. This study aimed to develop and internally validate a novel risk prediction model for ACN, with particular emphasis on incorporating gastric‐related indicators to enable integrated gastrointestinal tumor screening.

**Methods:**

Consecutive patients who underwent concurrent gastroscopy and colonoscopy for screening, symptomatic assessment, or surveillance purposes were recruited from the endoscopy center between January 1, 2021, and December 31, 2023. Comprehensive data including demographic characteristics, family history, *Helicobacter pylori (H. pylori)* infection status, and gastric histopathological results were collected. Patients with incomplete data were excluded a priori with no imputation. Candidate variables were selected using Elastic Net and LASSO regression to develop the prediction model. The model was trained on 80% of the data (*n* = 2045) and internally validated on the remaining 20% (*n* = 495).

**Results:**

Among 2540 participants [mean age 59.0 years; 1269 males (50.0%)], the overall prevalence of ACN was 13.1% (333/2540). The final Elastic Net model incorporated eight variables, with *H. pylori* infection as the strongest predictor. In the internal validation cohort, the model achieved an area under the curve (AUC) of 0.837 (95% CI: 0.781–0.892), with 75.8% sensitivity, 82.2% specificity, and 81.4% accuracy. Calibration metrics showed good agreement between predicted and observed ACN risks (Brier score = 0.103; calibration plots showed non‐significant deviation from perfect calibration). It significantly outperformed the Asia‐Pacific Colorectal Screening (APCS) score (AUC = 0.622), its revised edition (AUC = 0.589), and the Colorectal Tumor Prediction Score (AUC = 0.570) (all *p < 0.001*, DeLong's test). Using the model's optimal cutoff, 25.1% (124/495) of the internal validation cohort were stratified as high‐risk, with an ACN detection rate of 37.9%. The number of participants needed to screen (PNS) to detect one ACN case was only 3 in the high‐risk group, compared with 25 in the low‐risk group, thus demonstrating markedly improved screening efficiency.

**Conclusions:**

This study develops and internally validates a risk prediction model for ACN that integrates gastric‐related indicators, with *H. pylori* infection as the key predictor. The model exhibits promising retrospective risk stratification performance in patients undergoing concurrent gastroscopy and colonoscopy for screening, symptomatic assessment, or surveillance. It enables retrospective risk assessment during routine endoscopy, but its clinical utility for guiding prospective colonoscopy referral decisions requires further validation in prospective cohorts.

## 1 Introduction

Interval colorectal cancers (CRCs) account for up to 30% of all CRC cases [1], underscoring the need to optimize the identification of high‐risk patients who might benefit from intensified surveillance. Patients with *Helicobacter pylori (H. pylori)* infection, gastric polyps, and other gastric disorders have been shown to be at a significantly increased risk of colorectal cancer (CRC) [2, 3, 4, 5]. This association suggests that *H. pylori* infection could serve as a valuable risk enrichment marker to identify high‐risk subgroups that may benefit from targeted surveillance strategies. It has been widely reported that enhanced surveillance in this population may improve screening efficiency [6, 7].

The vast majority of patients with *H. pylori* infection, gastric polyps, or other gastric disorders alone will never develop CRC [8]. By accurately identifying the high‐risk subgroup, unnecessary surveillance procedures and associated healthcare costs could be reduced. Current risk‐stratification tools, including the Asia‐Pacific Colorectal Screening (APCS) score, APCS score revised edition, and Colorectal Tumor Prediction Score, have only suboptimal discrimination (with an AUC below 0.70) and show a small risk difference between low‐risk and high‐risk subgroups [9, 10]. Therefore, developing a risk‐stratification tool for individuals undergoing gastroscopy holds significant clinical value.

This study developed an endoscopic feature‐based prediction model for advanced colorectal neoplasia (ACN) by integrating variables including demographic characteristics and gastric‐related indicators (such as *H. pylori* infection), establishing a risk scoring scale for retrospective risk stratification of the study population.

## 2 Methods

### 2.1 Study Design and Population

We conducted a cross‐sectional study of consecutive patients who underwent gastroscopy and colonoscopy at the Affiliated Wuxi People's Hospital of Nanjing Medical University from January 1, 2021, to December 31, 2023. The study protocol was approved by the Wuxi People's Hospital Research Ethics Committee (KS202079).

Patients were included if they fulfilled the following criteria: age ≥ 18 years, with no gender restriction. Participants included asymptomatic individuals undergoing routine gastrointestinal screening, symptomatic patients with gastrointestinal complaints, and individuals undergoing colonoscopy for surveillance purposes.

Exclusion criteria were as follows: incomplete data; unsuccessful gastroscopy or colonoscopy; partial or total gastrointestinal resection due to gastric cancer, CRC, or other causes; hereditary polyposis syndromes (including Peutz‐Jeghers syndrome and familial adenomatous polyposis); and previous inflammatory bowel disease.

Missing data handling: patients with incomplete data were excluded a *priori*, with no missing data imputation applied.

### 2.2 Endoscopy Procedures and Histopathological Diagnosis

All endoscopists have worked for more than 5 years and performed over 5000 gastroscopies and 2000 colonoscopies. Before gastroscopy, patients were required to fast and received local anesthesia or mild sedation. The endoscope was introduced transorally, allowing the endoscopists to observe the gastric mucosa for any abnormalities in real time and perform *H. pylori* biopsies. During colonoscopy, patients were placed in the left lateral position. All colonoscopes were advanced into the ileocecal region. High‐definition colonoscopy (Olympus CV‐290) was used for examination. Colonoscopy quality indicators, including adenoma detection rate, withdrawal time, Boston Bowel Preparation Scale score [11], and endoscopist experience, were collected and stratified by indications (screening, symptomatic, surveillance).

The Department of Pathology examined the pathological specimens. They were fixed in 10% formaldehyde solution, embedded, sectioned, and stained with hematoxylin and eosin (H&E) for histological assessment and classification. H&E staining of the gastric mucosa specimens was performed to detect *H. pylori* infection. All pathological specimens were reviewed by two gastrointestinal pathologists at the Affiliated Wuxi People's Hospital of Nanjing Medical University. Notably, H&E staining only reflects the concurrent *H. pylori* infection status at the time of gastroscopy, and no data on infection duration, chronicity, or prior eradication history were collected.

According to the histopathological results, ACN was defined as colorectal cancer or advanced precancerous lesions identified on colonoscopy. Advanced precancerous lesions were defined as advanced adenoma (tubular adenoma ≥ 10 mm in the largest dimension, adenoma of any size with villous features, high‐grade dysplasia, or carcinoma in situ) or sessile serrated lesions at least 10 mm in the largest dimension [12, 13]. Gastric histopathological results included: non‐atrophic gastritis (normal mucosa or superficial gastritis), atrophic gastritis, gastric polyps (inflammatory polyps, hyperplastic polyps, fundic gland polyps, or adenomatous polyps), and gastric cancer or dysplasia [14, 15, 16, 17, 18, 19, 20].

### 2.3 Dataset Division

The study dataset was split into a training cohort (80%) and an internal validation cohort (20%) using stratified random sampling (random seed = 110) to ensure balanced distribution of key baseline characteristics (age, gender) between the two cohorts. This sampling method guaranteed representativeness of the validation cohort relative to the training cohort. The training cohort was used for variable selection and model development/parameter training; the internal validation cohort was used exclusively for independent performance evaluation, with no model retraining or parameter adjustment.

### 2.4 Variable Selection and Model Development

Variable selection was performed using data‐driven regularized regression models: Elastic Net (α = 0.5) and LASSO (α = 1) regression, which were chosen to prevent overfitting and identify the most parsimonious set of predictive variables (Figure 2). Prior to regression analysis, collinearity analysis was conducted for all candidate variables using the variance inflation factor (VIF); only variables with VIF < 10 were retained, confirming no significant multicollinearity between *H. pylori* infection and other gastric histopathological variables. The Elastic Net model was optimized via 10‐fold cross‐validation on the training cohort using the lambda.1se criterion (to select the most parsimonious model with performance within one standard error of the minimum cross‐validation error). The final Elastic Net model included eight predictive variables, and regression coefficients from the model were converted into a quantitative risk scoring system. The optimal risk stratification cutoff for high/low‐risk grouping was derived exclusively in the training cohort using Youden's index (maximizing sensitivity + specificity) and applied unchanged to the internal validation cohort to avoid over‐optimism. All analyses were performed in R software (version 4.4.1) using the glmnet package for regularized regression.

### 2.5 Model Validation and Performance Evaluation

Model validation was conducted using the independent internal validation cohort, with all analyses adhering to the TRIPOD statement (Transparent Reporting of a Multivariable Prediction Model for Individual Prognosis or Diagnosis).

Discrimination: Assessed via the area under the receiver operating characteristic (ROC) curve (AUC), with Bootstrap correction (500 iterations) applied to adjust for optimism and estimate the corrected AUC (Figure 4). DeLong's test was used to compare AUCs between the proposed model and three established risk scores (APCS score, APCS revised edition, Colorectal Tumor Prediction Score). Calibration: Evaluated using calibration plots (plotting predicted vs. observed ACN risks) and the Brier score (range: 0–1; lower values indicate better calibration). Calibration plots were fitted with a non‐parametric loess curve to visualize the agreement between predicted and observed risks, with a reference line representing perfect calibration. Clinical performance: Assessed using sensitivity, specificity, positive predictive value (PPV), negative predictive value (NPV), overall accuracy, and the number of participants needed to screen (PNS) to detect one ACN case. Subgroup analysis: Model performance was evaluated separately in pre‐specified subgroups stratified by colonoscopy indication. For each subgroup, we calculated the AUC with 95% confidence intervals (CIs), sensitivity, specificity, PPV, NPV. DeLong's test was used to compare AUCs between subgroups to assess performance consistency. All subgroup analyses were performed using the same model and cutoff derived from the training cohort, with no re‐calibration in subgroups.

All performance metrics are reported with 95% confidence intervals (CIs). The pROC package in R was used for ROC curve analysis, Bootstrap correction, and DeLong's test.

### 2.6 Risk Category Derivation and Clinical Utility

The optimal risk threshold for classifying high‐risk (HR) and low‐risk (LR) individuals was determined using Youden's index from the ROC curve. Clinical efficiency was assessed using the number of participants needed to screen (PNS) to detect one ACN case in each risk group. Given the retrospective cross‐sectional study design, the model currently enables retrospective risk stratification only. Prospective application to guide clinical decisions requires further validation in prospective cohorts.

### 2.7 Statistical Analyses

Continuous variables were summarized as mean ± SD or median (IQR) and compared using *t*‐tests or Mann–Whitney U tests. Categorical variables were expressed as frequencies (%) and compared using chi‐square or Fisher's exact tests. All analyses were performed using R software (version 4.4.1), with statistical significance set at *p* < 0.05 (two‐sided). The glmnet package was used for regularized regression, and the pROC package for ROC curve analysis and Bootstrap correction.

## 3 Results

### 3.1 Patient Characteristics

**FIGURE 1:**
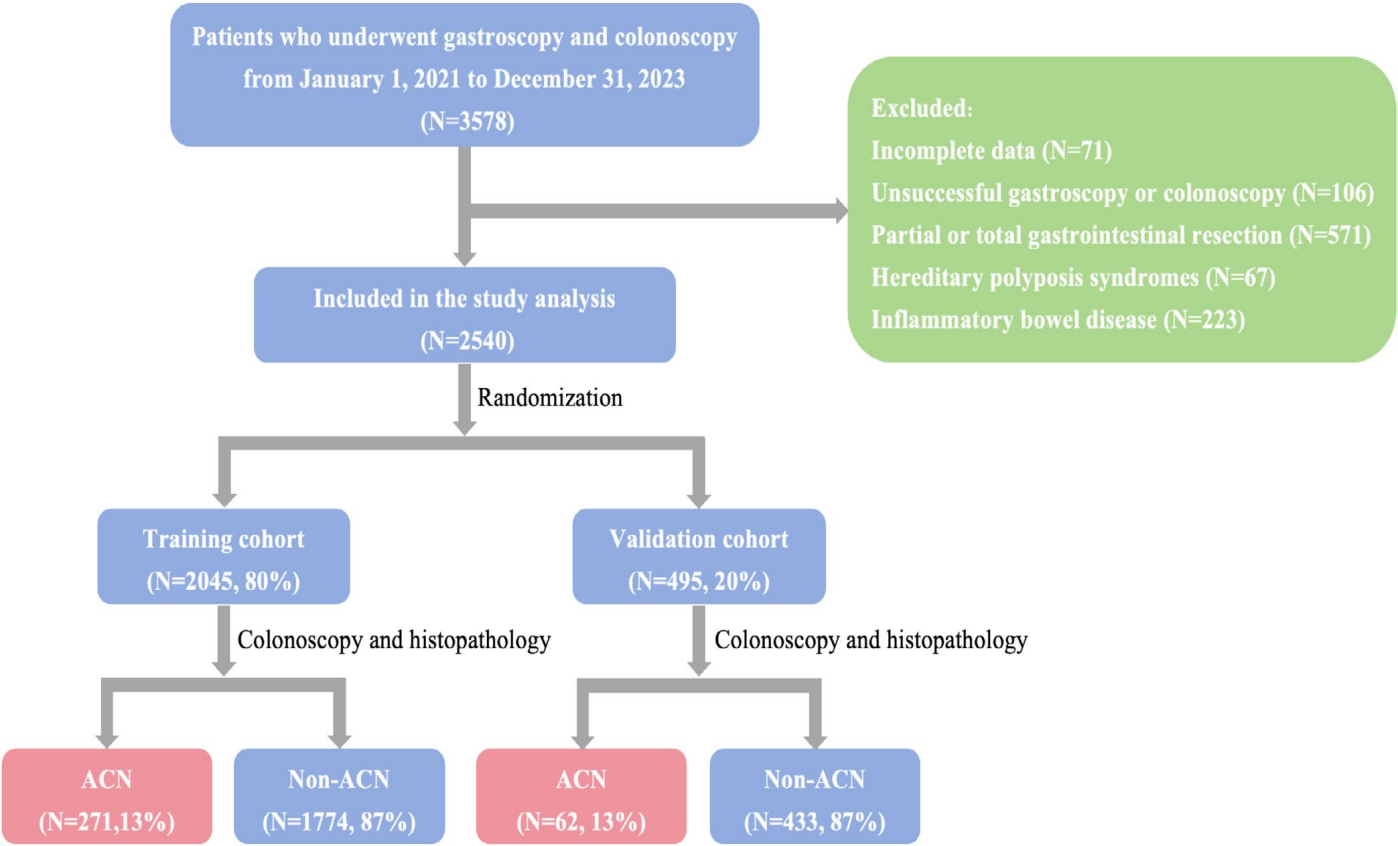
Flow chart of patients during the study period.

Figure 1 illustrated the participant selection process, with 2540 eligible individuals included in the final statistical analysis after applying the inclusion/exclusion criteria. Of these, 333 participants (13.1%) were diagnosed with advanced colorectal neoplasia (ACN), while the remaining 2207 (86.9%) comprised the non‐ACN group. The study population was then randomly divided into training cohort (*n* = 2045) and internal validation cohort (*n* = 495). Table 1 presented the characteristics of the 2540 participants included in the study, with the prevalence of *H. pylori infection* being 30.51% (775/2540).

**TABLE 1:** Characteristics of the training and validation cohorts.

Characteristic		Total	Training cohort	Validation cohort
		*N* = 2,540^**a**^	*N* = 2,045^**a**^	*N* = 495^**a**^
Gender	Male	1269 (49.96%)	1022 (49.98%)	247 (49.90%)
	Female	1271 (50.04%)	1023 (50.02%)	248 (50.10%)
Age (years)		59.02 ± 12.49	59.16 ± 12.49	58.44 ± 12.47
BMI^b^ (kg/m^2^)		23.64 ± 3.44	23.62 ± 3.39	23.71 ± 3.61
Hypertension	No	1523 (59.96%)	1240 (60.64%)	283 (57.17%)
	Yes	1017 (40.04%)	805 (39.36%)	212 (42.83%)
Diabetes	No	1924 (75.75%)	1551 (75.84%)	373 (75.35%)
	Yes	616 (24.25%)	494 (24.16%)	122 (24.65%)
Hyperlipidemia	No	2131 (83.90%)	1732 (84.69%)	399 (80.61%)
	Yes	409 (16.10%)	313 (15.31%)	96 (19.39%)
Fatty liver disease	No	2263 (89.09%)	1834 (89.68%)	429 (86.67%)
	Yes	277 (10.91%)	211 (10.32%)	66 (13.33%)
Coronary atherosclerotic heart disease	No	2324 (91.50%)	1874 (91.64%)	450 (90.91%)
	Yes	216 (8.50%)	171 (8.36%)	45 (9.09%)
Renal insufficiency	No	2241 (88.23%)	1823 (89.14%)	418 (84.44%)
	Yes	299 (11.77%)	222 (10.86%)	77 (15.56%)
Stroke	No	2256 (88.82%)	1824 (89.19%)	432 (87.27%)
	Yes	284 (11.18%)	221 (10.81%)	63 (12.73%)
Thyroid disease	No	2154 (84.80%)	1726 (84.40%)	428 (86.46%)
	Yes	386 (15.20%)	319 (15.60%)	67 (13.54%)
Chronic obstructive pulmonary disease	No	2526 (99.45%)	2033 (99.41%)	493 (99.60%)
	Yes	14 (0.55%)	12 (0.59%)	2 (0.40%)
Mental illness (including anxiety and depression)	No	2452 (96.54%)	1973 (96.48%)	479 (96.77%)
	Yes	88 (3.46%)	72 (3.52%)	16 (3.23%)
Viral hepatitis	No	2391 (94.13%)	1930 (94.38%)	461 (93.13%)
	Yes	149 (5.87%)	115 (5.62%)	34 (6.87%)
Rheumatoimmune disease	No	2389 (94.06%)	1923 (94.03%)	466 (94.14%)
	Yes	151 (5.94%)	122 (5.97%)	29 (5.86%)
Family history of CRC in a first‐degree relative	No	2179 (85.79%)	1758 (85.97%)	421 (85.05%)
	Yes	361 (14.21%)	287 (14.03%)	74 (14.95%)
Previous colonoscopy history	No	1852 (72.91%)	1489 (72.81%)	363 (73.33%)
	Yes	688 (27.09%)	556 (27.19%)	132 (26.67%)
Smoking	No	2372 (93.39%)	1910 (93.40%)	462 (93.33%)
	Yes	168 (6.61%)	135 (6.60%)	33 (6.67%)
Alcohol consumption	No	2425 (95.47%)	1955 (95.60%)	470 (94.95%)
	Yes	115 (4.53%)	90 (4.40%)	25 (5.05%)
Gastric histopathological results^c^	Non‐atrophic gastritis	1674 (65.91%)	1343 (65.67%)	331 (66.87%)
	Atrophic gastritis	340 (13.39%)	275 (13.45%)	65 (13.13%)
	Gastric polyps	406 (15.98%)	333 (16.28%)	73 (14.75%)
	Gastric cancer or dysplasia	120 (4.72%)	94 (4.60%)	26 (5.25%)
*Helicobacter pylori* infection	No	1765 (69.49%)	1427 (69.78%)	338 (68.28%)
	Yes	775 (30.51%)	618 (30.22%)	157 (31.72%)
Boston Bowel Preparation Scale	< 6	190 (7.48%)	154 (7.53%)	36 (7.27%)
	> = 6	2350 (92.52%)	1891 (92.47%)	459 (92.73%)
Endoscopist experience	Junior	372 (14.65%)	303 (14.82%)	69 (13.94%)
	Senior	2168 (85.35%)	1742 (85.18%)	426 (86.06%)
Indications	Screening	857 (33.74%)	694 (33.94%)	163 (32.93%)
	Symptomatic	674 (26.54%)	517 (25.28%)	157 (31.72%)
	Surveillance	1009 (39.72%)	834 (40.78%)	175 (35.35%)
Withdrawal Time		377.93 ± 142.87	377.52 ± 141.89	379.61 ± 146.99
Adenoma Detection Rate	Yes	898 (35.35%)	711 (34.77%)	187 (37.78%)
	No	1642 (64.65%)	1334 (65.23%)	308 (62.22%)
Advanced colorectal neoplasia	Yes	333 (13.11%)	271 (13.25%)	62 (12.53%)
	No	2207 (86.89%)	1774 (86.75%)	433 (87.47%)

^a^ Data shown as mean ± SD or *n* (%).

^b^ BMI, Body Mass Index.

^c^ Gastric histopathological results: Non‐atrophic gastritis (the histopathological report of the gastroscopy showed normal mucosa or superficial gastritis); Atrophic gastritis (the histopathological report of the gastroscopy showed chronic atrophic gastritis with or without intestinal metaplasia); Gastric polyps (the histopathological report of the gastroscopy showed inflammatory polyps, hyperplastic polyps, fundic gland polyps, or adenomatous polyps); Gastric cancer or dysplasia (the histopathological report of the gastroscopy showed gastric cancer, low‐grade intraepithelial neoplasia, or high‐grade intraepithelial neoplasia).

### 3.2 ACN Model Development and Validation

**FIGURE 2:**
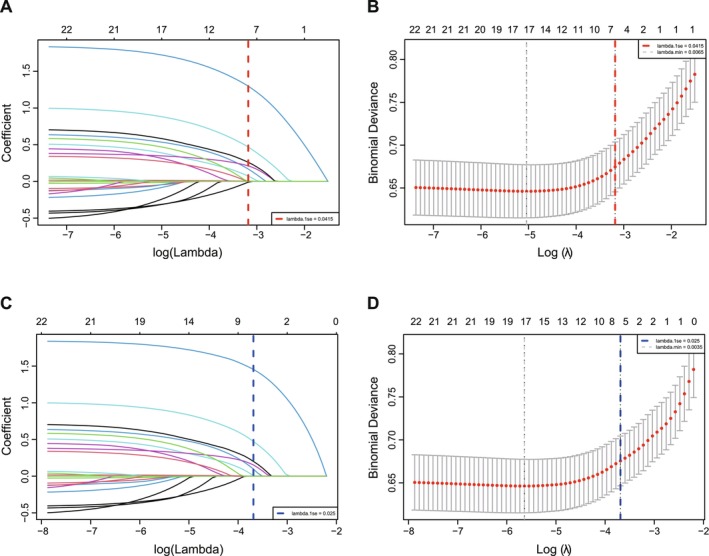
Coefficient path plot and cross‐validation error plot. A: Elastic Net Coefficient Path Plot; B: Elastic Net Cross‐Validation Error Plot; C: LASSO Coefficient Path Plot; D: LASSO Cross‐Validation Error Plot.

**FIGURE 3:**
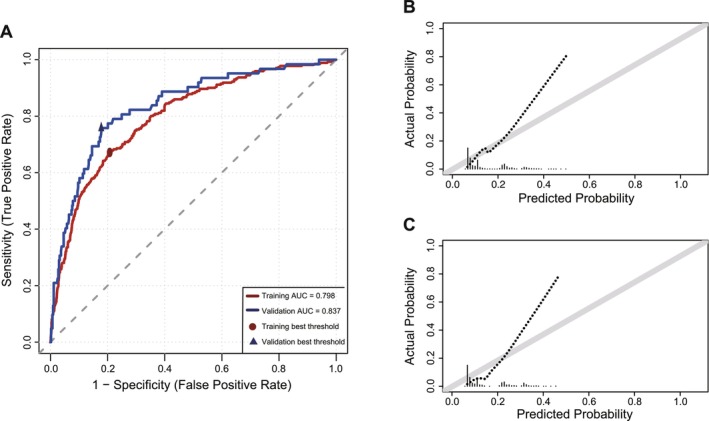
ROC and calibration curves of the training and validation cohorts. A: ROC curves of the training and validation cohorts; B: Training cohort calibration curve (Elastic Net); C: Validation cohort calibration curve (Elastic Net).

**FIGURE 4:**
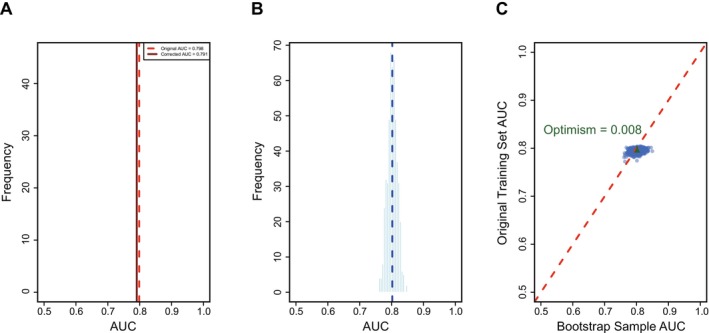
Bootstrap resampling AUC distribution plot. A: Bootstrap AUC distribution (Original Training Cohort); B: Bootstrap AUC distribution (Bootstrap Samples); C: Bootstrap optimism visualization.

Variable selection via Elastic Net and LASSO regression (Figure 2) identified eight independent predictive variables for inclusion in the final model (Tables 2 and 3), with no significant multicollinearity detected (all VIF < 5). *H. pylori* infection was the strongest predictor (coefficient = 1.2985, OR = 3.66), followed by previous colonoscopy history, alcohol consumption, smoking, family history of CRC, diabetes, gender, and age. In the training cohort, the model demonstrated good discriminative performance: AUC = 0.798 (95% CI: 0.770–0.827). Bootstrap correction (500 iterations) yielded a corrected AUC of 0.791 (95% CI: 0.785–0.802), indicating minimal optimism and high internal consistency (Figure 4). Calibration was excellent (Brier score = 0.098; calibration plot with no significant deviation from perfect calibration). In the internal validation cohort, the model maintained robust performance (Table 2): AUC = 0.837 (95% CI: 0.781–0.892), sensitivity = 75.8%, specificity = 82.2%, accuracy = 81.4%, PPV = 37.9%, and NPV = 96.0%. Calibration remained good (Brier score = 0.103, Figures 3 and 4), with the loess curve closely aligning with the perfect calibration reference line, indicating strong agreement between predicted and observed ACN risks.

**TABLE 2:** Performance metrics for the ACN model.

Performance metrics	Training cohort		Validation cohort	
	Elastic net	LASSO	Elastic net	LASSO
AUC^a^ (95% CI)	0.798 (0.770–0.827)	0.795 (0.766–0.824)	0.837 (0.781–0.892)	0.831 (0.776–0.887)
Sensitivity (%)	67.2	68.3	75.8	77.4
Specificity (%)	79.3	78.0	82.2	80.4
Accuracy (%)	77.7	76.7	81.4	80.0
Balanced accuracy (%)	73.2	73.1	79.0	78.9
Positive predictive value (%)	33.1	32.1	37.9	36.1
Negative predictive value (%)	94.1	94.1	96.0	96.1
F1 score	0.443	0.437	0.505	0.492
Best threshold	0.206	0.227	0.211	0.228

^a^ AUC, area under the receiver operating characteristic curve.

**TABLE 3:** Variable scale for the ACN model (Elastic Net).

Variable	Coefficient	Odds ratio	Importance ranking
*Helicobacter pylori* infection	1.2985	3.6637	1
Previous colonoscopy history	0.4682	1.5972	2
Alcohol consumption	0.2604	1.2975	3
Smoking	0.2118	1.2360	4
Family history of CRC in a first‐degree relative	0.1688	1.1838	5
Diabetes	0.0721	1.0747	6
Gender	−0.0105	0.9896	7
Age	0.0032	1.0032	8

**TABLE 4:** Comparison among four prediction models^a^.

	APCS score	APCS score revised edition	Colorectal Tumor Prediction Score	ACN model
AUC^b^ (95% CI)	0.622 (0.556–0.688)	0.589 (0.524–0.653)	0.570 (0.504–0.636)	0.837 (0.781–0.892)
*p* value (vs ACN Model)	< 0.001	< 0.001	< 0.001	—
Risk categories	3	3	2	2
Risk variables included	4	5	6	8
Low risk group [*n*, (%)]	92 (18.6%)	24 (4.8%)	332 (67.1%)	371 (74.9%)
Moderate risk group [*n*, (%)]	277 (56.0%)	348 (70.3%)	—	—
High risk group [*n*, (%)]	126 (25.5%)	123 (24.8%)	163 (32.9%)	124 (25.1%)
Calculation process	Easy	Easy	Easy	Easy

^a^ In validation cohort.

^b^ AUC, area under the receiver operating characteristic curve.

**TABLE 5:** Prevalence of ACN by risk category.

Risk model^a^	Risk category^c^	Total number^d^ *n*, (%)	ACN number^b^ *n*, (%)	Participants needed to screen^e^
		495 (100.0%)	62 (12.5%)	8
ACN Model	Low	371 (74.9%)	15 (4.0%)	25
	High	124 (25.1%)	47 (37.9%)	3
APCS score	Low	92 (18.6%)	1 (1.1%)	92
	Moderate	277 (56.0%)	33 (11.9%)	8
	High	126 (25.5%)	28 (28.2%)	5
APCS score revised edition	Low	24 (4.8%)	1 (4.2%)	24
	Moderate	348 (70.3%)	36 (10.3%)	10
	High	123 (24.8%)	25 (20.3%)	5
Colorectal Tumor Prediction Score	Low	332 (67.1%)	34 (10.2%)	10
	High	163 (32.9%)	28 (17.2%)	6

^a^ In validation cohort.

^b^ The prevalence rate of ACN.

^c^ In validation cohorts, there is a significant difference in the prevalence of ACN across the two risk categories.

^d^ Proportion relative to all participants of the validation cohort.

^e^ Defined as the number of participants who should undergo gastroscopy screening to identify one patient with ACN.

**TABLE 6:** Model performance stratified by colonoscopy indication.

Group	*n*	AUC (95% CI)	Sensitivity	Specificity	PPV	NPV
Full Cohort Subgroups						
Screening	857	0.821 (0.753–0.889)	74.3%	81.5%	36.8%	95.7%
Symptomatic	674	0.845 (0.772–0.918)	76.9%	83.1%	39.2%	96.3%
Surveillance	1009	0.832 (0.760–0.904)	75.2%	82.7%	38.5%	95.9%
Internal Validation Cohort						
Overall	495	0.837 (0.781–0.892)	75.8%	82.2%	37.9%	96.0%

### 3.3 Performance Comparison Among Four Prediction Models

Comparative analysis revealed that among the four prediction models assessed in the internal validation cohort, the ACN model showed the best discrimination (AUC = 0.837, 95% CI: 0.781–0.892). In contrast, APCS score [0.622 (0.556–0.688); *p* < 0.001 vs. ACN model, DeLong test], the APCS score revised edition [0.589 (0.524–0.653); *p* < 0.001 vs. ACN model, DeLong test] and the Colorectal Tumor Prediction Score [0.570 (0.504–0.636); *p* < 0.001 vs. ACN model, DeLong test] displayed significantly lower AUCs than the ACN model (Table 4). This advantage is attributed to the inclusion of gastric‐related indicators (e.g., *H. pylori* infection), which capture proximal gut microenvironment alterations not considered by existing models and serve as effective risk enrichment markers for ACN.

### 3.4 Risk Stratification and Clinical Efficiency

Using the optimal threshold, the ACN model stratified the internal validation cohort into high‐risk (*n* = 124, 25.1%) and low‐risk (*n* = 371, 74.9%) groups (Table 5). The ACN detection rate was 37.9% in the high‐risk group (PNS = 3) versus 4.0% in the low‐risk group (PNS = 25). This stratification outperformed existing models in terms of PNS and risk gradient (Table 5). Notably, all participants in this study underwent colonoscopy regardless of predicted risk, so the model currently provides retrospective risk stratification; prospective application to guide colonoscopy referral decisions is a key direction for future research.

### 3.5 Subgroup Performance by Colonoscopy Indication

Model performance was consistent across subgroups stratified by colonoscopy indication (screening, symptomatic, surveillance), with no significant differences in AUC between subgroups (*p* > 0.05, DeLong's test; Table 6). Screening subgroup (*n* = 857): AUC = 0.821 (95% CI: 0.753–0.889), sensitivity = 74.3%, specificity = 81.5%, PPV = 36.8%, NPV = 95.7%. Symptomatic subgroup (*n* = 674): AUC = 0.845 (95% CI: 0.772–0.918), sensitivity = 76.9%, specificity = 83.1%, PPV = 39.2%, NPV = 96.3%. Surveillance subgroup (*n* = 1009): AUC = 0.832 (95% CI: 0.760–0.904), sensitivity = 75.2%, specificity = 82.7%, PPV = 38.5%, NPV = 95.9%.

## 4 Discussion

In this study, we developed an enhanced prediction model for advanced colorectal neoplasia (ACN) by incorporating *H. pylori* infection. The new model achieved significant improvement in discrimination and expanded the risk gradient. To the best of our knowledge, this is the first ACN prediction model integrating gastric‐related indicators, enabling retrospective colorectal cancer risk stratification during gastroscopy across screening, symptomatic, and surveillance populations.

Several prediction models are available to estimate the risk of ACN, with the most widely used ones being the APCS score, its revised edition, and the Colorectal Tumor Prediction Score. The APCS score, developed by the Asia‐Pacific Working Group, is a simple and easy‐to‐use scoring calculator that includes age, sex, family history of CRC in a first‐degree relative, and smoking as risk factors, assigning a score to each parameter. It divides an individual's risk into low risk (LR), moderate risk (MR), and high risk (HR) levels to evaluate the likelihood of ACN. The revised APCS score adds BMI as an additional risk factor, while the Colorectal Tumor Prediction Score incorporates self‐reported diabetes to predict the overall risk of colorectal adenoma, advanced adenoma, and CRC in asymptomatic populations. However, the aforementioned ACN prediction models have only fair discrimination (AUC of less than 0.70) [21, 22], this limitation might stem from the fact that they solely incorporate patient‐related factors and that there is a small risk difference between low‐risk and high‐risk groups, indicating that a considerable number of patients might be missed in diagnosis. We hypothesized that incorporating additional risk factors that serve as effective risk enrichment markers would improve model discrimination and/or the risk gradient between groups compared with previous models, among which *H. pylori* infection is the most prominent one, serving as a risk enrichment marker rather than a deterministic causal factor for ACN. Numerous studies have confirmed the significant role of *H. pylori* infection [23, 24], gastric polyps [4, 25, 26], and other gastric disorders [27, 28, 29] in the development and progression of ACN. For example, a comprehensive meta‐analysis of 12 studies (*n* = 15,678) demonstrated a significant association between *H. pylori* infection and increased colorectal adenoma risk (OR = 1.34, 95% CI: 1.12–1.60), particularly pronounced in Asian populations (OR = 1.52) [23]. Coincidentally, researchers have demonstrated that the presence of gastric polyps was associated with an increased incidence of advanced colorectal neoplasia and CRC. However, although these studies demonstrated that gastric‐related indicators were potential risk enrichment markers for ACN development, current prediction models have failed to incorporate these gastric‐related factors into their algorithms.

Our model, which integrates *H. pylori* infection, achieved an AUC of 0.837 in the internal validation cohort, representing a substantial improvement over existing tools (ΔAUC > 0.2). Notably, *H. pylori* infection emerged as the strongest predictor, underscoring the potential role of gut microbiome and mucosal immunity in colorectal carcinogenesis. The odds ratio of *H. pylori* infection for ACN in this model was 3.66. This elevated OR is likely attributable to selection bias and indication‐related confounding inherent to the study population—consisting of patients undergoing concurrent gastroscopy and colonoscopy, who have a higher baseline prevalence of gastrointestinal abnormalities and thus represent a higher‐risk subgroup compared to the general population. Notably, the prevalence of *H. pylori* infection in our cohort was 30.51%, which is consistent with the infection rate reported in previous studies [30], indicating that the proportion of *H. pylori* infection in our study does not deviate from the reasonable range reported in the relevant literature. While this elevated OR does not invalidate the model's internal validity (it performs well in the study cohort), it raises important considerations regarding the model's transportability, calibration, and predictor effect stability in other settings (e.g., the general population, asymptomatic screening cohorts without upper gastrointestinal symptoms). Further external validation in diverse populations is needed to adjust for this effect, refine the *H. pylori* risk estimate, and confirm the model's generalizability beyond the study cohort.

From a clinical perspective, the model's risk stratification demonstrated high efficiency: only 3 high‐risk individuals needed to be screened to detect one ACN case, compared with 25 in the low‐risk group. This represents a marked improvement over conventional models, such as the APCS score, which required 92 screenings in its low‐risk category to identify one case. The high NPV (96.0%) also suggests the potential to reduce unnecessary colonoscopies in low‐risk patients, but this hypothesis requires prospective validation.

A notable finding in our model is that previous colonoscopy history was a positive predictor of ACN, which may appear counterintuitive given its established protective role in CRC prevention for the general population. This variable in our study reflects underlying surveillance indications (e.g., pre‐existing gastrointestinal polyps, chronic gastritis) rather than routine preventive colonoscopy in asymptomatic low‐risk individuals; participants with a previous colonoscopy history in this cohort have a higher baseline risk of gastrointestinal neoplasia, which explains its positive predictive value in the model. This context‐dependence underscores the need to interpret predictors within the specific study population and indicates the model is best suited for screening, symptomatic, and surveillance populations undergoing concurrent gastroscopy and colonoscopy.

Our study has several limitations. First, this research is based on a single‐center, retrospective cross‐sectional design and solely incorporates colorectal cancer screening data from the Wuxi region, which may limit the generalizability of the model. Due to regional differences in factors such as sanitation conditions, dietary habits, and living environments, the prevalence of *H. pylori* infection, the spectrum of gastric pathology, and the incidence of advanced colorectal neoplasia may vary significantly across different regions. Such geographic specificity may restrict the applicability of the model to other populations and geographical areas. Second, the study lacks external validation. The 20% split‐sample cohort used in this study is an internal validation cohort derived from the same center, time period, and population, and external validation in multiple centers and diverse populations is needed to confirm the model's robustness. Meanwhile, we are currently conducting a multicenter prospective study to further validate the model's applicability in diverse populations and regions. Third, the detection of *H. pylori* infection in this study was conducted using H&E staining rather than more sensitive methods such as immunohistochemistry, which may lead to misclassification of some false‐negative cases as uninfected, thereby underestimating the true effect of *H. pylori* as a risk factor [31, 32]. Furthermore, H&E staining only assesses contemporaneous *H. pylori* infection status at the time of gastroscopy, and no data on infection duration, chronicity, or prior eradication history were collected; thus, it is impossible to determine whether *H. pylori* represents a long‐term exposure relevant to colorectal carcinogenesis or a contemporaneous marker correlated with other risk factors. Additionally, despite validation in an internal cohort, the model still carries a certain degree of misclassification risk, as some cases of advanced colorectal neoplasia were categorized as low‐risk, potentially delaying timely intervention. Fourth, the model currently enables only retrospective risk stratification; all participants underwent colonoscopy regardless of predicted risk, so the model has not been prospectively applied to guide clinical colonoscopy referral decisions. Its clinical utility in real‐world screening scenarios, including its ability to reduce unnecessary colonoscopies and improve ACN detection rates, requires prospective validation. Fifth, while the study adheres to the TRIPOD statement, future studies should collect additional data (e.g., OLGA/OLGIM staging of gastric mucosa) to more accurately assess the incremental value of gastric mucosal pathological changes in predicting ACN risk.

In conclusion, we developed a new prediction model for advanced colorectal neoplasia (ACN) by incorporating gastric‐related indicators. Comprehensive analyses showed that our ACN model enhanced the precision of retrospective risk stratification for high‐risk individuals in the population undergoing concurrent gastroscopy and colonoscopy, allowing retrospective gastric/colorectal cancer risk assessment during routine endoscopy. Given the retrospective cross‐sectional design and lack of prospective validation, the model currently cannot be used to guide clinical colonoscopy referral decisions or adjust screening intervals. It provides a theoretical basis for improving the cost‐effectiveness of gastrointestinal tumor screening programs, but its real‐world clinical utility requires further prospective validation in diverse populations.

## Author Contributions

Xujin Chen: conceptualization, investigation, data curation, formal analysis, writing‐original draft. Dehua Tang: methodology, software, validation, visualization. Ningjing Gao: investigation, resources. Muhan Ni: investigation, data curation. Peng Yan: investigation, validation. Zhanwen Ji: investigation (Endoscopic assessment). Xiang Xin: data curation, formal analysis support. Cheng Yang: supervision, project administration, writing‐review and editing. Lei Wang: supervision, resources, writing‐review and editing. Qiang Zhan: supervision, writing‐review and editing.

## Data Availability

The data that support the findings of this study are available on request from the corresponding author. The data are not publicly available due to privacy or ethical restrictions.
